# Severe acute respiratory infection caused by swine influenza virus in a child necessitating extracorporeal membrane oxygenation (ECMO), the Netherlands, October 2016

**DOI:** 10.2807/1560-7917.ES.2016.21.48.30416

**Published:** 2016-12-01

**Authors:** Pieter L A Fraaij, Enno D Wildschut, Robert J Houmes, Corien M Swaan, Christian J Hoebe, H C C de Jonge, Paulien Tolsma, Isme de Kleer, Suzan D Pas, Bas B Oude Munnink, My V T Phan, Theo M Bestebroer, R Shanty Roosenhoff, Jeroen J A van Kampen, Matthew Cotten, Nancy Beerens, Ron A M Fouchier, Johannes H van den Kerkhof, Aura Timen, Marion P Koopmans

**Affiliations:** 1 Department of Viroscience Erasmus MC, Rotterdam, The Netherlands; 2 Department of Pediatrics, Subdivision Infectious diseases and Immunology, Erasmus MC – Sophia, Rotterdam, The Netherlands; 3 Intensive Care and Department of Pediatric Surgery, Erasmus MC-Sophia, Rotterdam, The Netherlands; 4 Centre for Infectious Disease Control-National Institute for Public Health and the Environment, Bilthoven, The Netherlands; 5 Department of Sexual Health, Infectious Diseases and Environmental Health, Public Health Service South Limburg, Geleen, The Netherlands; 6 Faculty of Health, Medicine and Life Sciences Department of Medical Microbiology, Maastricht Infection Center (MINC),School of Public Health and Primary Care (CAPHRI),Maastricht University Medical Center (MUMC+), Maastricht, The Netherlands; 7 Gemeentelijke Gezondheidsdienst Rotterdam-Rijnmond, Rotterdam, The Netherlands; 8 Gemeentelijke Gezondheidsdienst Brabant zuidoost, Eindhoven, The Netherlands; 9 Department of Paediatrics, Subdivision of pulmonary medicine, Erasmus MC – Sophia, Rotterdam, The Netherlands; 10 Wageningen Bioveterinary reseach- Wageningen University and Research, Lelystad, the Netherlands; Wageningen University and Research, Wageningen the Netherlands

**Keywords:** Influenza, Severe, Swine, Children, ECMO

## Abstract

In October 2016, a severe infection with swine influenza A(H1N1) virus of the Eurasian avian lineage occurred in a child with a previous history of eczema in the Netherlands, following contact to pigs. The patient’s condition deteriorated rapidly and required life support through extracorporeal membrane oxygenation. After start of oseltamivir treatment and removal of mucus plugs, the patient fully recovered. Monitoring of more than 80 close unprotected contacts revealed no secondary cases.

We here report a patient with severe acute respiratory infection as a result of swine influenza virus (SIV) infection in the Netherlands.

## Case description

A school-aged patient with a previous history of mild eczema developed a respiratory tract infection in October 2016, a couple of days after visiting a pig farm. The child had entered the pigsty but had not been in direct contact with pigs. Despite early prescription of antibiotics by the general practitioner the child’s clinical situation rapidly deteriorated. Within three days after onset of disease the child was transferred to a paediatric intensive care unit (PICU) for non-invasive ventilation support and intensive monitoring. Despite these efforts, the patient deteriorated further and was intubated in order to start mechanical ventilation. Bronchoscopy following intubation revealed large amounts of highly viscous mucus in the airways. Efforts to remove this mucus failed to improve ventilation. Mechanical ventilation became increasingly complex and it was decided to initiate veno-venous extracorporeal membrane oxygenation (ECMO) and to transfer to a quaternary PICU. Due to ECMO, blood oxygenation was secured and extensive bronchoscopy could be performed, during which topical DNAse (Dornase alpha, Pulmozyme, Roche) was instilled to decrease viscosity and facilitate removal of obstructing mucus plugs. On the following day, bronchoscopy was repeated and additional mucus was removed.

In the days following these procedures, the patient improved rapidly. ECMO was discontinued five days after start and the patient could be extubated. For the entire duration of hospitalisation, the patient had received broad-spectrum antibiotics, although all bacterial cultures remained negative. Throat swabs had been collected at initial admission and tested positive for influenza A virus, of which the quaternary PICU was informed on the day after the patient transfer. Oseltamivir treatment (60 mg twice daily) was started hours after initiation of ECMO and transport. It was continued for a total of 7 days when a nasal swab tested negative for influenza virus. At the time of submission of this report, the child was recovering well.

## Virological results

The initial diagnostic routine was limited to testing for the influenza A virus matrix gene, without subtyping. In view of the severe course of illness, the child was resampled for repeated testing including typing of the haemagglutinin (HA) gene by quantitative real-time PCR for H1 (seasonal and pdm2009), H3, H5, H7 and H9. All typing PCRs were negative.

We determined the full virus genome sequences of a cell culture isolate derived from a respiratory tract sample using Illumina MiSeq. All gene segments (GenBank accession numbers KY250316-KY250323) were 97–98% and 98–100% identical at, respectively, nucleotide and amino acid level to publicly available SIV sequences from the Netherlands (GISAID accession numbers EPI639351, EPI639914, EPI639917, EPI639930, EPI640657, EPI640912, EPI641210, EPI641215). The gene segments were all of the Eurasian avian A(H1N1) SIV lineage that has been circulating in pigs since 1979 [1]. Pigs at the farm visited by the patient tested positive for the same SIV (curation of full genome sequence data is in progress). The virus isolate from the patient, A/Netherlands/3315/2016, was sensitive to oseltamivir and zanamivir by NA-star neuraminidase inhibitor resistance detection assay (Applied Biosystems, Nieuwerkerk aan den IJssel, The Netherlands).

## Public health measures

Zoonotic influenza is a notifiable disease in the Netherlands. Following confirmation of the zoonotic SIV infection, the national and relevant municipal public health authorities were notified and a teleconference was organised to decide on measures. The risk for human-to-human transmission was considered very low, given the enzootic presence of swine influenza viruses and the fact that zoonotic infections are seldom diagnosed.

In order to detect human-to-human transmission at an early stage, it was decided to contact all individuals that had been in close direct contact with the patient without wearing personal protective equipment, and monitor them for symptoms of possible SIV infection (cough, fever or conjunctivitis) for 10 days after exposure. In total, more than 80 contacts were monitored. These included the patient’s family members living in the same household, persons living and working on the pig farm, and healthcare workers who cared for the patient without wearing personal protective equipment (i.e. before the influenza diagnosis). Six contacts developed mild respiratory symptoms including cough, coryza and conjunctivitis during the monitoring period but all tested negative for influenza A virus.

According to the international health regulations, this case has been notified to the European Union Member States and the European Centre for Disease Prevention and Control (ECDC) through the Early Warning and Response System (EWRS) and to the World Health Organization (WHO).

## Discussion

Incidental cases of human infection with SIV have been reported worldwide since the late 1950s. Most of these were in individuals exposed to pigs. Apart from one isolated incident in military barracks in the United States (US), sustained and efficient human- to- human transmission had not been documented before 2009, when an influenza virus of swine origin triggered the first influenza pandemic of the 21st century. Indeed it is speculated that pigs may serve as a mixing vessel for the development of a pandemic influenza strain [2-5]. In addition, SIV infections account for roughly one third of all laboratory-confirmed zoonotic influenza events reported in the scientific literature [3]. This may be a gross underestimation of the actual number as there are no typical signs and symptoms that distinguish SIV infections in humans from those caused by seasonal influenza viruses [4]. Indeed several sero-epidemiological studies suggest that SIV infection in people with occupational swine exposure is common [6-8]. In the US, there is a routine surveillance for swine influenza in pigs, and 400 patients with a swine influenza infection have been reported through this system since 2005 [9]. Our case shows that careful assessment of airway disease in individuals exposed to pigs continues to be important, especially considering the importance of starting of antiviral treatment early.

## Conclusion

We here describe that transmission of SIV to humans, though rare, can occur and cause severe disease requiring life support through ECMO. Monitoring of people in direct contact and not wearing personal protective equipment revealed no secondary cases.
